# Supplementary material for: Causal Relationship between Aging and Anorexia Nervosa: A White-Matter-Microstructure-Mediated Mendelian Randomization Analysis
Source: Biomedicines. 2024 Aug 16;12(8):1874. doi: 10.3390/biomedicines12081874 (PMC11351342; doi:10.3390/biomedicines12081874)
Supplement: Supplementary file 1 [file biomedicines-12-01874-s001.zip › Supplementary Figure/Figure S9/Leaveoneout plot of MR analysis for ubm-b-1926 to AN.pdf]

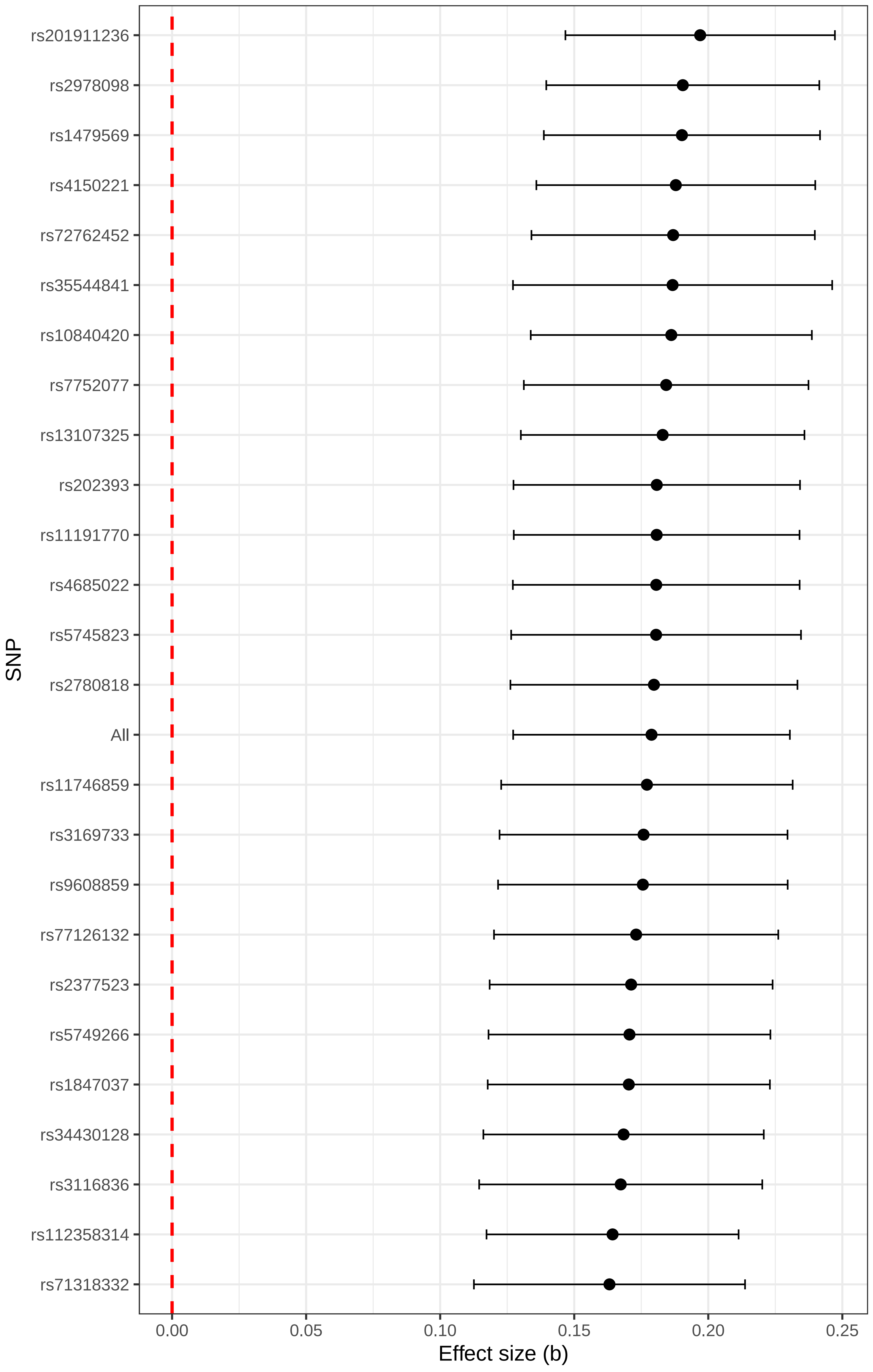
rs201911236
rs2978098
rs1479569
rs4150221
rs72762452
rs35544841
rs10840420
rs7752077
rs13107325
rs202393
rs11191770
rs4685022
rs5745823
rs2780818
All
rs11746859
rs3169733
rs9608859
rs77126132
rs2377523
rs5749266
rs1847037
rs34430128
rs3116836
rs112358314
rs71318332
SNP
0.00
0.05
0.10
0.15
0.20
0.25
Effect size (b)
